# Could formaldehyde induce mutagenic and cytotoxic effects in buccal epithelial cells during anatomy classes?

**DOI:** 10.4317/medoral.21492

**Published:** 2016-12-06

**Authors:** Diego-Coelho Lorenzoni, Leon-Penido Pinheiro, Haniel-Serpa Nascimento, Cristiani-Sartorio Menegardo, Ronara-Gerhardt Silva, Willian-Grassi Bautz, José-Fernando Henriques, Karla-Loureiro Almeida-Coburn, Letícia-Nogueira da Gama-de-Souza

**Affiliations:** 1 Postgraduate student. Department of Orthodontics, Bauru Dental School, University of São Paulo, Bauru, Brazil; 2 Undergraduate student. Department of Morphology, Federal University of Espírito Santo, Vitória, Brazil; 3 Professor. Department of Morphology, Federal University of Espírito Santo, Vitória, Brazil; 4 Professor. Department of Orthodontics, Bauru Dental School, University of São Paulo, Bauru, Brazil

## Abstract

**Background:**

Due to increased formaldehyde exposure, carcinogenic to humans, several researches have been studying the potential toxicity and the safe levels for human beings. The aim of this study was to investigate mutagenicity and cytotoxicity in buccal epithelial exfoliated cells (BEC) of students subjected to formaldehyde (FA) during anatomy classes.

**Material and Methods:**

BEC were collected periodically from 17 volunteers of undergraduate programs, who had participated in practical anatomy classes, before and after FA exposure. Cells were stained according to Feulgen method and then micronucleus test was applied. A total of 1,500 cells were assessed per individual in this study for the micronucleus frequency and other parameters of cytotoxicity.

**Results:**

There was statistically significant increase in number of micronucleated BEC after FA exposure (after 1 month *p*=.034 and after 3.5 months *p*=.017). However, FA exposure caused no significant increase in other nuclear alterations closely related to cytotoxicity (*p*≥.05).

**Conclusions:**

FA induced mutagenicity during anatomy classes. Cell death increased, but it was not statistically significant. Efforts have to be made to improve air quality and reduce exposures during anatomy classes.

**Key words:**Carcinogens, formaldehyde, micronucleus tests, mutagenicity tests.

## Introduction

Formaldehyde (methanal) (FA) is the most simple and reactive aldehyde. In its natural state, formaldehyde is a gas, and due to its high water solubility, aqueous solutions of the reactant can be produced (1). It is a compound with a wide range of industrial applications and is commonly used in anatomy and pathology laboratories as a fixative to preserve anatomical specimens. It is also a product of normal human metabolism (1,2).

At room temperature, FA is quickly volatilised to a pungent and suffocating colourless gas with a distinct odour, which can be recognised by humans at concentrations below 1 ppm (3). Inhalation of FA is thought to lead to deposition and/or absorption, mainly in the oral and nasal mucosa, regions that are in direct contact with the gas (4). Presently, the International Agency for Research on Cancer classifies FA as carcinogenic to humans, based on sufficient evidence in humans and in experimental animals (5). Due to increased exposure, which occurs in factories, hospitals and universities, several researches have been studying the potential toxicity and the safe levels for human beings (6). At the same time, efforts have been made to improve air quality and reduce exposures during anatomical dissections and anatomy classes (7).

Genetic damage caused by genotoxic agents, such as FA, can be measured using biomonitoring tests, and the micronucleus (MN) test is a very reliable assay for evaluating mutagenicity. This test is based on the formation of micronuclei from particles of chromatin material that, due to chromosome breakage or spindle dysfunction, do not migrate to the poles during anaphase and are not incorporated into the telophase nuclei of the dividing cell and result in the formation of one or more small satellite nuclei in the cytoplasm of the daughter cells (8).

Micronucleus test has a well-established protocol that is performed in human peripheral blood lymphocyte cultures. MN evaluations are made in buccal epithelial exfoliated cells (BEC) as well, and this test is considered to be the least invasive method available to measure DNA damage in humans (9). Due to its ability to assess the activity of many chemical or physical carcinogenic and mutagenic agents in situ, the MN test in the BEC is the choice of many recent human biomonitoring studies: alcohol (10), tobacco (11), mouthrinses (12) and radiographs (13).

Formaldehyde’s ability to generate MN is recognized in the literature. However, there are still questions about the frequency and amount of MN (3). It is important to emphasise that, although dispersed FA could be rapidly metabolised, the molecules that enter the cytoplasm of mitotically active cells could cause DNA damage, resulting in an increased frequency of MN (4).

Although the focus of previous studies was to analyse the well-recognised cytological abnormalities mentioned above, variables such as exposure time, gas concentration at the time of exposure, and other related factors (for example smoking, alcohol intake, acute infections and severe allergies) still vary greatly among the available references (1,3). This suggests a lack of standardisation and systematisation of the collection and analysis of previous data (14). Furthermore, most studies focused on chronic FA exposure, and this study aimed to analyse the presence of nuclear changes in epithelial cells from the buccal mucosa of individuals who were exposed to FA only during 3.5 months.

## Material and Methods

-Subjects 

The subjects of this study included a total of 17 (8 males and 9 female; 19.5 years old + 1.73) health students who attended the biological sciences program and the dentistry program at the Federal University of Espírito Santo, Vitória, Brazil. All selected students were enrolled in a weekly human anatomy class, with a total of 30 (n=11) to 90 (n=6) hours per semester of laboratory practise. It is important to emphasise these practical lessons occurred in the same anatomy laboratory for both courses, so the students came into contact with FA gas in similar conditions. The sanitary policy of the university states that measurement of toxic gases in laboratories that use FA as a fixative and preservative of specimens must be performed. An independent company was hired to perform the analysis, and the rate of FA gas exposure in the anatomy laboratory was 0.73 ppm. The procedures were in accordance with the ethical standards on human experimentation and were approved by the Ethics in Research Committee of Federal University of Espírito Santo, Vitória, Brazil (number 275/10-CEP) and it is in accordance with The Code of Ethics of the World Medical Association (Declaration of Helsinki) for experiments involving humans. All participants provided a written informed consent after receiving a full explanation of the study objectives and structure.

Before the first practise session, a questionnaire was given to establish a sample profile. The questionnaire was also a tool to screen for exclusion criteria, which included: history of cancer, prior chemotherapy and/or radiotherapy, diabetes, smoking, heavy alcohol consumption, previous contact with FA, radiographic exams in the chest and neck in the last 16 days, use of anabolic steroids, chronic bronchitis, asthma, chronic rhinitis, nasal solution use, active orthodontic treatment, and medication use.

-Micronucleus test in buccal epithelial exfoliated cells

At first, exfoliated buccal mucosa cells were collected immediately before FA exposure (t0). Then 1 month (t1) and 3,5 months (t2) after the initial contact with this gas, they were collected again. After rinsing the mouth with tap water, cells were obtained by scraping the right/left cheek mucosa with a moist wooden spatula. Cells were transferred to a tube containing saline solution, fixed in 3:1 methanol/acetic acid and dropped onto pre-cleaned slides. Later, the air-dried slides were stained using the Feulgen/Fast Green method (15) and examined under a light microscope at x400 magnification to determine the frequency of micronucleated cells (MNC). The cells were scored directly on the slides from each patient for each sampling time (t0, t1 and t2).

-Data analysis

**Figure 1:**
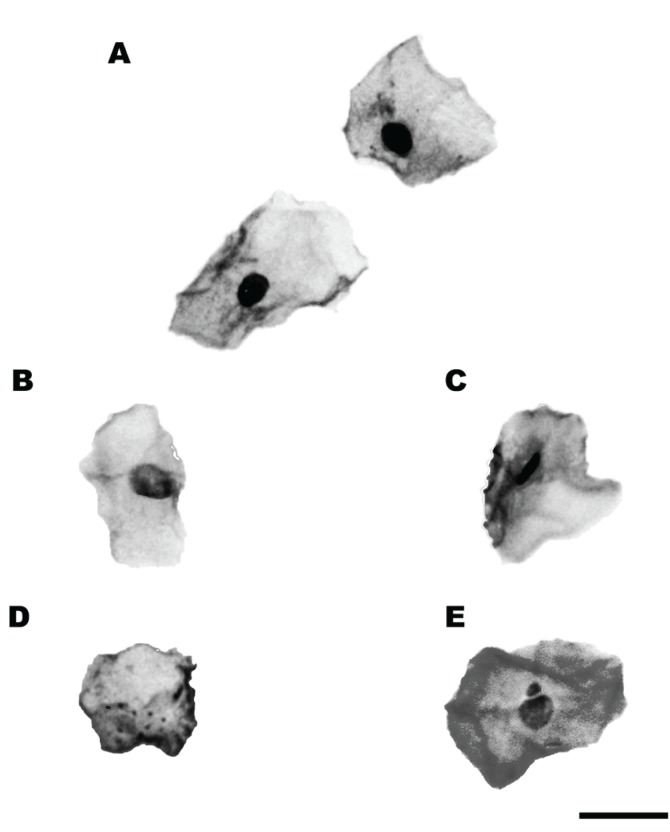
Microscopic features of nuclear aspects in oral epithelial cells. (A) Normal cells; (B) Karyorrhexis; (C) Pyknosis; (D) Karyolysis; (E) Micronucleus. Feulgen/Fast Green stain. Scale bar = 20 µm.

Two calibrated researchers analyzed the slides. The micronucleated cells (measure of DNA damage) were scored according to the criteria described by Sarto *et al * (16): Micronucleus was identified taking into consideration the following conditions: Intact nucleus and cytoplasm; diamater of 1/3 of the main nucleus; same staining and texture as the main nucleus and micronucleus was in the same focal plane as the main nucleus. For cytotoxicity, the following nuclear alterations were considered as described by Tolbert *et al.* (17): pyknosis, karyolysis and karyorrhexis (Fig. 1 a-d ). A total of 1,500 cells were assessed per person in this study for the micronucleus frequency and other parameters of cytotoxicity. The results were calculated by assessing % of altered cells only. Results are expressed in percentages (%). Similar analyses were established in previous published studies (13,18). The processing procedures and analysis were performed at the LUCCAR Lab, Histology Sector, Morphology Department, Federal University of Espírito Santo, Vitória, Brazil. All of the slides were masked to avoid evaluator-related bias in interpretation of the results.

-Statistical methods

The concordance between investigators was evaluated by Fleiss’Kappa test. Kolmogorov-Smirnov test was applied to confirm the validity of normality assumption of all variables. The paired-samples t test was used to compare the frequencies of nuclear alterations related to cytotoxicity and mutagenicity, before and after FA exposure. The level of statistical significance was set at 5%.

To evaluate concordance between evaluators, digital pictures of six hundred BEC from this research were used. These cells were numbered and classified according to their nuclear characteristics: normal, pyknosis, karyolysis, karyorrhexis and MNC, and the Fleiss’ Kappa test was applied to investigate the concordance between the two investigators.

## Results

According to Fleiss’ Kappa test, the concordance between the two investigators was almost perfect (Kappa value = 0.8203).  Table 1 shows frequency of MNC (mutagenicity) and other nuclear alterations (cytotoxicity) in students submitted to FA during anatomy classes. Before FA contact, the mean frequency of MNC was 0.05%. This frequency increased significantly after 1 month (0.11%; *P*=.034) and 3.5 months (0.16%; *P*=.017) of contact with the gas, showing mutagenic effect. An increase in other nuclear alterations (karyorrhexis, pyknosis, and karyolysis) was observed after FA exposure, but it was not statistically significant (*P*≥.05), evidencing no cytotoxicity. These data are summarized in  Table 1. None of the evaluated students were exposed to other known genotoxic agents.

**Table 1:**
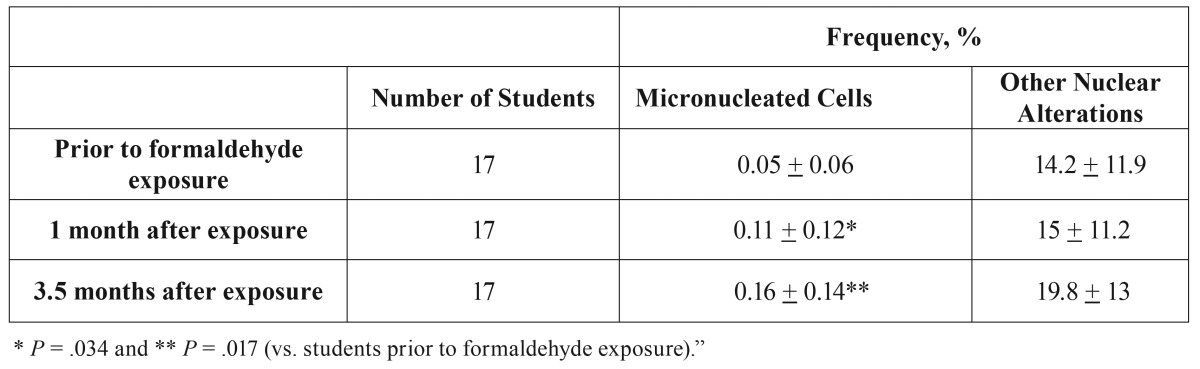
Frequency (%) of micronucleated cells and other nuclear alterations (karyorrhexis, pyknosis, and karyolysis) in students exposed to formaldehyde. Values are means + standard deviation (SD).

## Discussion

Buccal epithelial cells represent a preferred target site for early genotoxic events induced by carcinogenic agents entering the body via inhalation and ingestion (9). Add to that the knowledge that 90% of all oral human cancers originate from epithelial cells (19). These facts highlight one advantage of the MN assay, an *in vivo* exam that elucidates the effects of toxic agents directly on a target tissue, the buccal epithelium. The limited cost, ease of counting, person-time required and precision obtained from scoring large numbers of cells improve the popularity of this non-invasive method (13).

Damages that lead to the formation of micronuclei take place in the basal layer of epithelial tissue where cells undergo mitosis. The rapid turnover of epithelial tissues brings the cells to the surface where they exfoliate (20). In general, cells take 7-16 days to emerge to the surface and exfoliate (21). For this reason, exfoliated oral mucosa cells were collected immediately before FA exposure and after 1 and 3.5 months of contact with the toxic gas. This period allowed time for the basal layer that was exposed to FA to mature and be collected when exfoliated. Several staining methods have been used, although DNA specific stains are preferred for staining nuclei, micronuclei, and other nuclear anomalies in buccal exfoliated cells. Feulgen-Fast Green staining is the choice of many investigators because of its DNA specificity and a clear transparent appearance of the cytoplasm, which enables easy identification of micronuclei. Micronucleus test with DNA non-specific stains in buccal exfoliated cells have been used (22), but it is suggested that cellular structures resembling micronucleus could be stained, such as keratohyalin granules or bacteria, which can lead to false positive results. Actually, DNA specific stains like Feulgen-Fast Green, used in this investigation, are the correct choice for micronucleus tests in buccal exfoliated cells according to the most important researches in this field, like Human Micronucleus Project (9).

Human biomonitoring studies in buccal cells involve several confounding factors such as age, lifestyle, oral hygiene (e.g., mouthrinse utilization), dental health, smoking and alcohol (9). These factors were controlled in our study. Moreover, each patient was considered to be his own control. So, any effect of other genotoxic agents must have been present in the first cell count. Therefore, potential differences between the prior to and after gas exposure can be attributed to FA. Some authors have pointed towards a relationship between age and MN occurrence (23), whereas others have not (8,24). Due to the homogeneity in sample, it was not possible to correlate the frequency of MNCs with age in this setting.

Several studies have investigated local genotoxicity of FA inhalation in humans. An important literature review about genotoxic effects of FA measured by the micronucleus test in exfoliated epithelial cells was published in 2006, and described that it is still not possible to assess the local genotoxicity of FA in humans and to draw meaningful conclusions about the relation between dose-effect and risk estimation, especially because of large variability and quality of published studies (14). After that, a research done under strictly controlled conditions revealed no mutagenic characteristics on buccal exfoliated cells, after 21 days of FA exposition (25).

On the other hand, significant DNA damage in lymphocytes and buccal cells after occupational FA exposure in factories, pathology and anatomy laboratories and students of Mortuary Science is described (1,26-28), showing that the damage extent was directly proportional to the duration of exposure (1,27). Duration of exposure to formaldehyde really seems to influence the onset of mutagenic effects on buccal cells. After 21 days of exposure, a research showed that there was not genotoxic effect (25). However, after 1 and 3.5 months of contact, this research showed that the mutagenicity not only occurred after the first month, but also increased after that. These data reinforce the importance of improving air quality and reduce exposures during anatomical dissections and anatomy practical classes (7). It is important to stress that occupational exposure to FA evaluated by other investigations (1,26-29) presented greater contact time with formaldehyde and its concentration in the air was higher than those found for anatomy students, which differentiate them from this research. In this investigation, students (n=17) with different exposure times to FA (30 or 90 hours per semester) were included in the same group. If they had been separated according to exposure time, this research would have two small groups, which would undermine the analysis. It is important to stress that two-thirds of included students had lower contact time with the FA (30 hours, n=11) and, nevertheless, mutagenic effect has been identified. This suggests that even with reduced contact time, FA is harmful.

With regards to the rate of MN, the literature reports a wide variation in the incidence of MN. Comparing the results of this study with others studies (1,30), MN was detected in a lower frequency before or after contact with FA. This fact could be explained by socioeconomic and cultural differences, since it is known that lifestyle and environmental exposures can affect MN formation (9). Another limitation is that most of the previous studies were conducted with occupational exposure individuals, which is quite different from the profile of the individuals in this study. Another important question is that the occurrence of MNC is small and its distribution is not homogeneous in the sample. These facts explain the large standard deviation commonly found in similar researches and highlights the need for counting a large number of cells in this kind of research to minimize errors (10,12,13,18,24).

Researchers have called attention to nuclear changes other than MN that characterize cellular death and may increase the sensitivity of tests to detect genotoxicity (12,13,17). It is known that repeated exposure to cytotoxic agents can result in chronic cell injury, compensatory cell proliferation, hyperplasia, and, ultimately, tumor development. These cytotoxic/non-genotoxic agents act by interfering with the molecules intimately involved in cell growth and cell death. Increased cell proliferation appears to be a unifying feature of epigenetic carcinogens. Proliferation may increase the risk of mutations within target cells and may also be important in the selective clonal expansion of initiated cells (31). However, there are no previous reports of FA cytotoxicity by the MN test in buccal cells. Thus, cytotoxic effects were investigated through the frequencies of karyorrhexis, karyolysis, and pyknosis. Cell death increased after 1 and 3.5 months of FA exposure, but it was not statistically significant. The limited number of volunteers may have masked the statistical significance of this increase.

To summarize, according to micronucleus test, mutagenicity was induced by the contact with formaldehyde during anatomy classes and this effect increased with time. The cell death increased after formaldehyde exposure, but it was not statistically significant. An increase in the number of students assessed could show cytotoxic effects as well. Efforts should be done to improve air quality and reduce formaldehyde exposure during anatomical dissections and anatomy classes. In addition, more elaborated researches in this important field should be performed to confirm these findings, with a greater sample and standardization of the exposure time to FA.
